# Astrocytic regulation in hippocampal CA2 mediates the impact of sleep deprivation on spatial working memory

**DOI:** 10.3389/fnsys.2026.1744203

**Published:** 2026-02-17

**Authors:** Jingang He, Yunshuang Ye, Jun Fang, Jie Wang, Hoiyin Cheung

**Affiliations:** 1 School of Life Science and Technology, Harbin Institute of Technology, Harbin, China; 2 Department of Neurosurgery, Songjiang Research Institute, Shanghai Key Laboratory of Emotions and Affective Disorders, Songjiang Hospital Affiliated to Shanghai Jiao Tong University School of Medicine, Shanghai, China; 3 Department of Anesthesiology, Ruijin Hospital, Shanghai Jiao Tong University School of Medicine, Shanghai, China

**Keywords:** astrocytes, hippocampus CA2, metabolic kinetics, sleep deprivation, spatial working memory

## Abstract

**Introduction:**

Chronic sleep deprivation (CSD) is closely associated with significant mood disorders, such as anxiety and depression, and may lead to spatial memory impairment. Spatial memory is a cognitive function closely linked to the hippocampus, with the CA2 region playing a critical role in memory processing. However, the specific mechanisms by which the CA2 region contributes to spatial memory impairment induced by sleep deprivation remain unclear. This study hypothesizes that CSD impairs spatial memory by affecting the metabolic function of astrocytes in the hippocampal CA2 region.

**Methods:**

The study used 7-week-old C57BL/6J mice to establish a CSD model via the multi-platform water environment method (MPT). Functional magnetic resonance imaging (fMRI), including ALFF and ReHo analyses, was employed to assess functional changes in brain regions. Metabolic dynamics were studied using 13C-labeled glucose and sodium acetate to evaluate the metabolic states of neurons and astrocytes, respectively. Additionally, chemogenetic manipulation (via AAV viral vectors) was used to modulate the activity of astrocytes in the CA2 region, and spatial memory function was assessed through Y-maze behavioral tests.

**Results:**

CSD leads to functional abnormalities in the hippocampal CA2 region and spatial memory impairment in mice, as evidenced by increased ALFF and ReHo values in fMRI and decreased performance in the Y-maze test. Additionally, CSD induces metabolic dysregulation and calcium signaling abnormalities in CA2 astrocytes. Inhibition of calcium signaling exacerbates memory impairment, whereas activation of astrocytes can reverse this effect.

**Conclusion:**

Metabolic dysfunction and calcium signaling abnormalities in astrocytes of the hippocampal CA2 region are key mechanisms underlying spatial memory impairment caused by CSD. Activation of CA2 astrocytes can restore memory function, providing a novel therapeutic target for cognitive deficits associated with sleep disorders.

## 1 Introduction

Sleep deprivation (SD) is a state of insufficient sleep that results from a variety of factors and triggers a cascade of alterations in emotional regulation, learning and memory, immune function, and more. As societal pressures escalate and the pace of life accelerates, SD has become a significant global concern (Ng and Tan, 2005; Kolla et al., 2020). Chronic sleep deprivation (CSD) is closely associated with marked mood disturbances, such as anxiety and depression, and has been implicated in impairments of spatial working memory (Aquino and Schiel, 2023). Spatial working memory, a process intricately linked to the hippocampus, is essential for the encoding and retrieval of spatial information. The hippocampus, a critical structure in memory consolidation, plays a pivotal role in the processing and integration of spatial data through its interactions with cortical and hypothalamic regions (Parent et al., 2011). Recent studies have shown that SD impairs spatial working memory by modulating neurotransmitter dynamics, synaptic plasticity, and protein expression within the hippocampus (Wilson and McNaughton, 1994; Chee and Chuah, 2008). These biochemical alterations may disrupt hippocampal function, particularly within the dentate gyrus and CA1 regions, leading to deficits in both the formation and consolidation of spatial memories (Meier et al., 2020; Konakanchi et al., 2022; Oliva et al., 2023). The CA2 region of the hippocampus serves as a crucial intermediary in memory processing, with essential roles in both social and spatial memory (Oliva et al., 2023; Alexander et al., 2016). Notably, emerging evidence suggests that the CA2 region is involved in the consolidation of spatial memories during sleep (Piskorowski and Vivien, 2018; Kay et al., 2016). However, the precise role of the hippocampal CA2 region in the disruption of spatial working memory following SD remains to be fully elucidated.

In the field of brain function research, blood oxygen level-dependent functional magnetic resonance imaging (BOLD-fMRI) has emerged as a prominent technique, encompassing both task-related and resting-state fMRI (rs-fMRI) studies (Guo et al., 2024; Lv et al., 2018). Within the rs-fMRI domain, two technologies, amplitude of low-frequency fluctuations (ALFF) and regional homogeneity (ReHo), are widely used due to their robust reproducibility and clear physiological relevance. These measures provide insights into local brain activity, although they do not directly assess functional connectivity between distant brain regions (Cao et al., 2024). Although ALFF and ReHo analyses often yield similar outcomes, they offer complementary perspectives: ALFF serves as an indicator of neural activity, while ReHo reflects the degree of regional coordination. The combined application of these indices enables a more nuanced understanding of functional alterations in specific brain regions, underscoring the value of this approach in investigating the impact of sleep deprivation (SD) on spatial working memory (Yao et al., 2023; Lee et al., 2013).

On the other hand, chronic sleep deprivation (CSD) disrupts cerebral energy metabolism and neurotransmitter dynamics, leading to widespread neural dysfunction. Central to this disruption is the altered interaction between neurons and astrocytes, which impairs energy utilization and metabolite production across key brain regions, including the cortex, midbrain, and thalamus (Zhu et al., 2023a). Under physiological conditions, astrocytes maintain synaptic homeostasis through the glutamate-glutamine cycle-a process essential for recycling glutamate and preventing neuronal over excitation (Bellesi et al., 2017; Halassa and Haydon, 2010). CSD, however, compromises this cycle, resulting in deficient glutamate clearance, aberrant glutamine synthesis, and risk of excitotoxicity (Andersen et al., 2021; Mander et al., 2017). Since astrocytes supply neurons with metabolic substrates (e.g., lactate) via this cycle, their dysregulation directly undermines neuronal energy support, ultimately contributing to memory deficits (Andersen et al., 2021; Xie et al., 2013).

This study combines functional magnetic resonance imaging (fMRI) with metabolic dynamic analysis to demonstrate that CSD-induced impairments in spatial working memory are associated with metabolic dysfunctions in astrocytes within the hippocampal CA2 region, accompanied by disruptions in spontaneous calcium signaling. Inhibition of the inositol trisphosphate (IP3) pathway, a key second messenger in spontaneous calcium signaling in CA2 astrocytes, results in spatial memory deficits in mice. In contrast, activation of astrocytes within the hippocampal CA2 region facilitates the recovery of spatial memory in these animals. This study try to reveal novel insights into the hippocampal CA2 region's contribution to CSD-induced memory deficits, opening new avenues to comprehend the impact of sleep disorders on spatial working memory.

## 2 Methods

### 2.1 Animals

All animal procedures were approved by the Animal Ethics Committee of the Innovation Academy for Precision Measurement Science and Technology, Chinese Academy of Sciences (Approval No.: APM22022A), and conducted in accordance with the Animal Research: Reporting of *In Vivo* Experiments (ARRIVE) guidelines. The study utilized 7 week old male C57BL/6J mice (*n* = 56), sourced from the Hubei Provincial Center for Disease Control and Prevention (Wuhan, China). Mice were housed in groups of five per cage under a 12 h light-dark cycle, with room temperature maintained at 25 ± 1 °C, and food and water provided ad libitum. After a 1 week acclimatization period, the mice were randomly assigned to either the control or CSD group.

#### 2.1.1 Chronic sleep deprivation (CSD) model

**Figure 1:**
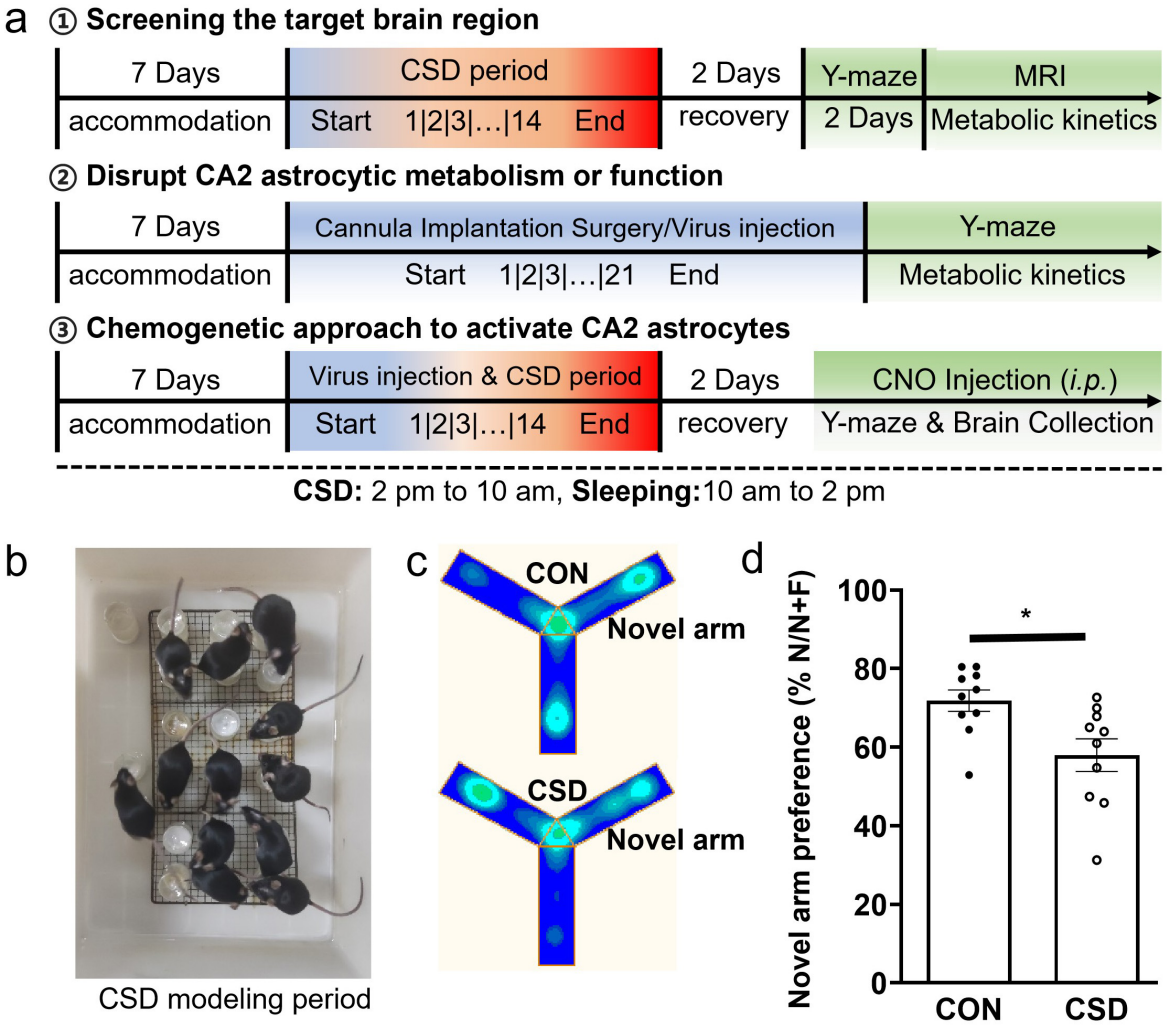
Chronic sleep deprivation impairs short-term spatial memory in mice. **(a)** Diagram of experimental design. **(b)** Enhanced multi-platform water environment real-time map. **(c)** Heatmap of Y-maze behavioral trajectory. **(d)** Comparison and sleep deprivation groups: Y-maze experiment results. CON: Control group; CSD: Chronic sleep deprivation group; *n* = 10 mice per group. ^*^p < 0.05.

CSD is induced using the modified water environment multiple platform technique (MPT), which specifically targets rapid eye movement (REM) sleep (Zhu et al., 2023b) (Figure 1a). The underlying principle of MPT is based on the significant reduction in muscle tone during REM sleep, rendering mice susceptible to intermittent awakenings when they come into contact with or are submerged in water (Zager et al., 2007). This technique enables the selective deprivation of REM sleep without disrupting other sleep stages. The SD apparatus consists of a 40 × 28 × 19 cm box housing 20 cylindrical platforms, each positioned 1 cm above the water surface. Each platform has a diameter of 2.5 cm and a height of 5.0 cm (Figure 1b). After a 7 day acclimation period, the experimental animals underwent a 14 day CSD protocol. During this period, the mice in the CSD group were placed in the SD chamber from 2:00 PM to 10:00 AM the following morning, resulting in a total of 20 h of SD. Subsequently, they were returned to their original cages for sleep recovery from 10:00 AM to 2:00 PM, amounting to 4 h of recovery sleep. Mice in the control group were similarly housed in the SD chamber from 2:00 PM to 10:00 AM, but a stainless steel mesh was installed above the chamber, allowing the mice to move freely and sleep on the mesh. They were also returned to their original cages from 10:00 AM to 2:00 PM18.

#### 2.1.2 fMRI experiments

fMRI data acquisition was performed using a 7.0 T scanner (Bruker, Ettlingen, Germany). Mice were initially anesthetized with 4%-5% isoflurane and then maintained under anesthesia with 1.0-1.5%. The animals were positioned on an animal bed equipped with a water-circulation heating system for magnetic resonance scanning. During image acquisition, the heads of the mice were securely fixed under the collector using earplugs and a tooth bar to ensure image integrity. Respiratory rate was continuously monitored and maintained between 70 and 90 breaths per min. A 20 cm transmission coil and a 10 mm surface coil were employed for imaging, with T2-weighted high-resolution scans used for structural imaging. Blood oxygenation level-dependent (BOLD) signals were acquired using a single spin-echo planar imaging sequence, with a repetition time (TR) of 1,500 ms, echo time (TE) of 14 ms, 210 repetitions, and a total scan duration of 10 min 30 s. All MRI scans were conducted between 9:00 AM and 5:00 PM to ensure consistency across all animals.

#### 2.1.3 Brain fixation with head focused microwave irradiation

Metabolic dynamics studies often employ 13C-labeled glucose or sodium acetate to assess neuronal or astrocytic functions (Guo et al., 2022). To decrease the effects of endogenous glucose, animals are fasted for 15-18 h prior to experimentation. On the day of the experiment (between 8:00 AM and 12:00 PM), following the fasting period, mice were briefly anesthetized with isoflurane, and a PE10 infusion cannula was inserted into the tail vein. The mice were then allowed a 30-minute recovery period in their home cage. The opposite end of the PE10 tube was connected to an infusion pump via a swivel joint. After 30 mins, a bolus of [1-13C] glucose or [2-13C] sodium acetate was intravenously administered over 2 mins. Following infusion, animals were allowed to move freely in their cage. After an additional 30 mins, the mice were anesthetized with an overdose of isoflurane and euthanized via decapitation (Guo et al., 2021). The heads were rapidly collected, and the brains were immediately subjected to microwave fixation (0.75 kW for 14 s) to terminate metabolic processes (Liu Y. et al., 2017). The brain was then dissected, with hippocampal regions identified according to the Allen Mouse Brain Atlas (3^rd^ edition). Samples were weighed and stored at −80 °C for subsequent analysis.

#### 2.1.4 Tissue extraction

The frozen brain tissue was transferred into a 2 mL EP tube, and 200 μL of HCl/methanol solution was added. The tissue was then homogenized using a tissue homogenizer for 90 s. Next, 800 μL of ethanol was introduced, and the mixture was homogenized and centrifuged for 10 mins. This procedure was repeated twice, after which the supernatant was collected and lyophilized using a centrifugal drying device followed by a freeze-drying vacuum dryer. The lyophilized product was dissolved in 600 μL of D2O buffer, containing 5mM 3-(trimethylsilyl) propionic acid-2,2,3,3-d4 sodium salt (TMSP) as the internal standard, and 0.2 M Na2HPO4 and NaH2PO4 at pH 7.2. The solution was vortexed and centrifuged at 13,000 rpm for 10 mins, and approximately 550 μL of the supernatant was transferred into a 5 mm NMR tube for subsequent NMR analysis.

#### 2.1.5 Data collection for [1H-13C] NMR

The prepared NMR tube was placed into the 500 MHz liquid magnetic resonance scanner. On the computer interface, tuned the sample, locked the field, homogenized the magnetic field, and calibrated the 90-degree excitation pulse to ensure accuracy during measurement. Once these steps were completed, initiated the POCE sequence. The POCE sequence could measure the metabolite content of 12C + 13C and 12C-13C, with a spectral width set to 16 ppm and a chemical shift range from −3 ppm to 13 ppm.

#### 2.1.6 Viral constructs

The following viral vectors were obtained from Brain Case Biotechnology Co., Ltd. (Shenzhen, China) for this study: AAV2/8-gfABC1D-hM3Dq(Gq)-mCherry-ER2-WRPA-PE, AAV2/5-gfABC1D-shRNA-IP3R2, AAV5rAAV-gfABC1D-jGCaMP7b, and AAV9rAAV-CaMKII α-ChR2-WrPa. All viral vectors were aliquoted and stored at −80 °C until further use. Unless otherwise specified, the viral titer for AAV injections was maintained at >5 × 10^12^ viral particles per milliliter.

#### 2.1.7 Stereotaxic surgery

Seven-week-old mice were lightly anesthetized with a single intraperitoneal injection of sodium pentobarbital (10 mg/ml, 50 mg/kg) and subsequently secured in a stereotactic frame (RWD Life Science, Shenzhen, China) using shatter-resistant ear bars to minimize discomfort. The scalp was incised along the midline, and symmetrical cranial openings were created with a microdrill fitted with a 0.5mm bit. Glass micropipettes for AAV microinjection were fabricated using a P-97 micropipette puller (Sutter Instrument Company, USA), with a tip diameter of 10–20 μm. These micropipettes were primed with silicone oil and connected to a microinjection pump (RWD Life Science, Shenzhen, China) to ensure degassing. The AAV solution was injected into the CA2 region at the following coordinates relative to bregma (anterior-posterior, AP; lateral-medial, ML; dorsal-ventral, DV; all units in millimeters): CA2: AP−2.92, ML ±3.40, DV−3.21. A volume of 0.2 μL was injected bilaterally at a rate of 0.03 μL/min. The micropipette was retained in place for 10 mins post-injection to allow for adequate diffusion. Mice were allowed to recover for 21 days before behavioral and other assessments. Post-experiment, the injection site was examined to assess mCherry expression, verifying injection accuracy and efficiency. For intracranial infusion experiments, cannulae (500 μm diameter, 5 mm length, RWD Life Science, Shenzhen, China) were placed above CA2 at coordinates AP = −2.92 mm, ML = ± 3.4 mm, DV = −3.11 mm. These cannulae were fixed with dental cement and secured with skull screws. The cannula covers (5.5 mm length) were applied post-surgery to prevent infection, and experiments commenced after a 2 week recovery period.

#### 2.1.8 Intracranial drug infusions prior to Y-maze test

Intracranial drug injections were performed 30 mins prior to the Y-maze test. During the procedure, the mouse's head was briefly restrained, and the stainless steel blocker was removed to facilitate the insertion of the injection cannula into the guide cannula. The injection cannula was advanced 0.5 mm beyond the tip of the guide cannula. A microinjection syringe, pre-filled with silicone oil to remove air, was connected to a microinjection pump (RWD Life Science, Shenzhen, China). fluorocitric acid (1 μL) was injected into the unilateral CA2 region at a rate of 100 nl/s on both sides. The syringe was left in place for 30 s to allow for adequate diffusion of the injected substance.

### 2.2 Data processing

#### 2.2.1 ALFF and ReHo

ALFF is defined as the sum of the amplitude of low-frequency fluctuations in the power spectrum of each voxel's signal, reflecting neural activity and representing the amplitude of spontaneous low-frequency oscillations in the BOLD signal. To minimize the effects of low-pass filtering on resting-state data, unfiltered data are used to calculate the ALFF value. A Fourier transform is applied to the unfiltered resting-state MRI signals to convert them into the frequency domain, and the ALFF value is derived by averaging the power spectrum within the 0.01-0.1 Hz frequency range (Zhang et al., 2023; Wang et al., 2021). ReHo is defined as the coherence coefficient between a specific voxel and its 27 neighboring voxels, reflecting the temporal coherence between the time series of a given voxel and its adjacent voxels (Dai et al., 2023). This metric requires resampling long TR data to match the spatial resolution of short TR data. Prior to computation, the resting-state time series undergoes high-pass filtering (>0.01 Hz) and low-pass filtering (<0.1 Hz).

#### 2.2.2 fMRI data processing

Raw T2-weighted and echo planar images are converted to NIFTI format using Bru2anz software (Bruker, Germany). The fMRI data are then preprocessed with ANTs software (Advanced Normalization Tools), which includes steps for denoising, time correction, head motion correction, smoothing, and registration. Following preprocessing, the data undergo bandpass filtering (0.01 to 0.1 Hz), and signals from white matter and cerebrospinal fluid are regressed. Finally, individual spatial configurations of ALFF and ReHo are computed.

#### 2.2.3 NMR data processing

For metabolic profiling, the extracted brain tissues were snap-frozen in liquid nitrogen and subsequently homogenized in ice-cold methanol/water (2:1, v/v). The mixture was centrifuged at 12,000 g for 15 mins at 4 °C, after which the supernatants were collected, lyophilized, and reconstituted in D2O containing 0.01% TSP for internal referencing. NMR spectra were acquired on a 600 MHz NMR spectrometer (Bruker, Germany) using a standard NOESY pulse sequence. All spectra were processed using TopSpin software (v3.5), including Fourier transformation, manual phasing, and baseline correction. Metabolite concentrations were then quantified by integrating peak areas relative to the TSP signal and normalized to the respective tissue weights.

For ^13^C enrichment analysis, the processed spectral data were further imported into the custom-developed Matlab software NMRSpec (Liu Y. et al., 2017) for spectral alignment and integration of chemically relevant peaks, with detailed peak information referenced to previous studies (Liu T. et al., 2017; Liu et al., 2020). Notably, the imported data included two hydrogen spectra: one representing the sum of ^12^C and ^13^C metabolites and the other corresponding to the difference between them, where the spectral difference reflects the relative concentration of 2 × ^13^C metabolites, and the integration results were automatically exported as target files for subsequent ^13^C enrichment calculation.

### 2.3 Y-maze behavioral test

Spatial working memory was assessed using the Y-maze spontaneous alternation test. The apparatus consisted of three identical arms (40 cm long, 9 cm wide, and 16 cm high) positioned at 120° angles. Distinct visual cues (geometric shapes) were placed on the walls surrounding the maze to facilitate spatial orientation. During the test, each mouse was placed at the center of the maze and allowed to explore freely for 8 mins. An entry was recorded when all four paws of the mouse entered an arm. A spontaneous alternation was defined as consecutive entries into all three different arms (e.g., ABC, BCA). The alternation percentage was calculated as: [number of alternations/(total number of arm entries - 2)] × 100. The maze was thoroughly cleaned with 75% ethanol between trials to eliminate olfactory cues.

### 2.4 Pharmacological and chemogenetic manipulations

For pharmacological inhibition of astrocytic metabolism, fluorocitric acid (FC, 10 nmol in 0.5 μL saline) was microinjected into the CA2 region 30 mins prior to behavioral testing. For chemogenetic activation, AAV-gfABC1D-hM3Dq-mCherry (0.3 μL) was bilaterally injected into the CA2. After 3 weeks of viral expression, Clozapine N-oxide (CNO, 1 mg/kg, i.p.) was administered 30 mins before the Y-maze test to activate the hM3Dq receptors. Control groups received saline or AAV-gfABC1D-mCherry injections respectively.

### 2.5 Statistical analysis

Data analysis was conducted using Matlab and ANY-MAZE software, with results presented as mean±SEM (standard error of the mean). Metabolic differences among various metabolites were assessed using ANOVA followed by Tukey's *post hoc* test. For behavioral data, differences between two groups were evaluated using independent samples *t*-test, while differences among multiple groups were analyzed using ANOVA. Statistical significance was set at *P* < 0.05. A significance level of *P* < 0.05 was adopted, with statistical significance indicated as ^*^P < 0.05, ^**^P < 0.01, and ^***^P < 0.001. Results with no statistical significance are labeled as N.S (non-significant).

## 3 Results

### 3.1 Chronic Sleep Deprivation (CSD) impairs spatial working memory

Following a 14 day period of CSD and a subsequent 2 day recovery phase, mice were subjected to the Y-maze test. Initially, the animals were positioned at the maze's starting point and allowed to explore two of the three arms, paths one and two. Driven by innate curiosity, the mice typically chose the novel arm while remembering the previously explored paths, enabling them to efficiently identify the unvisited arm (path three). This study utilized the Y-maze paradigm to assess short-term memory retention, specifically focusing on spatial working memory (Figure 1c). The results demonstrated a marked impairment in the ability of chronically sleep-deprived mice to identify the correct path, compared to controls [t (18) = 2.88, P = 0.01] (Figure 1d), corroborating findings from previous studies (Onaolapo et al., 2015; Griffin et al., 2023). These data further validate the Y-maze served as a reliable and effective tool for evaluating spatial working memory.

### 3.2 Abnormalities of regional function in hippocampal CA2 region of CSD mice

As described previously, the combined use of ALFF and ReHo analyses in functional MRI co-marking provides a direct screening of target brain regions with functional alterations. We employed this method to investigate the mechanisms by which CSD impacts spatial working memory through functional changes in the brain. Following CSD, mice first underwent a Y-maze behavioral test, followed by a functional MRI scan to gather data. Although we acknowledge the presence of subtle numerical variations between the left and right CA2 regions, we utilized bilateral regions of interest (ROIs) for the final statistical comparisons to enhance the signal-to-noise ratio (SNR) and ensure the robustness of our findings. This is a standard and widely accepted practice in rodent fMRI research (Pan et al., 2015).

**Figure 2:**
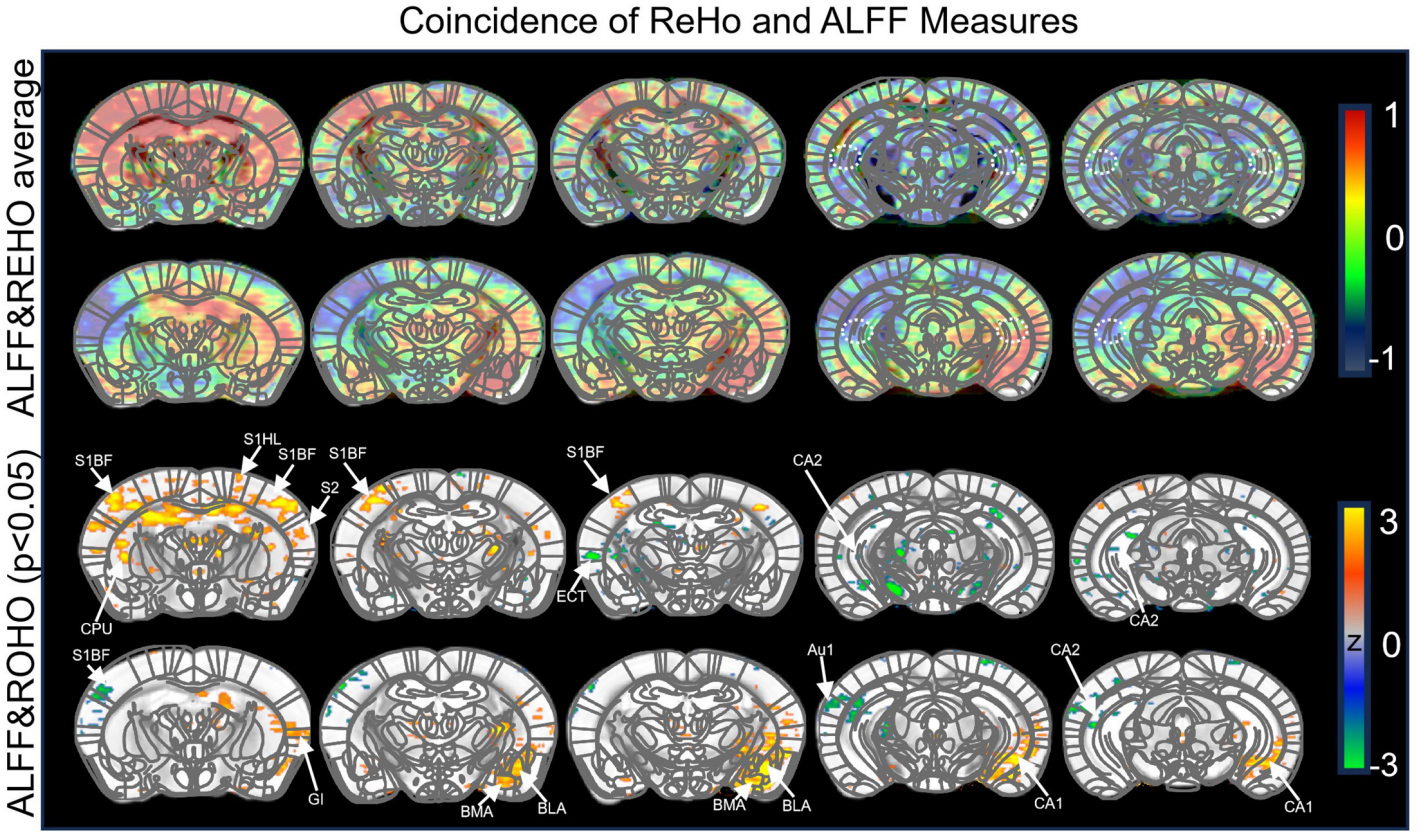
Comparison of low-frequency amplitude and regional homogeneity between control and chronic sleep-deprived groups. The first and third rows represent the mean values of low-frequency amplitude and the z-value differences, respectively. The second and fourth rows show the mean values of regional consistency and z-values, with the region of interest limited to the hippocampal CA2 and SuM areas. The following abbreviations are used for the respective brain regions: S1BF: Primary somatosensory cortex (barrel field); S2: Secondary somatosensory cortex; S1HL: Primary somatosensory cortex, hindlimb region; CPU: Caudate nucleus (striatum); CA1: Hippocampal CA1 region; GI: Gigantocellular reticular nucleus; BLA: Basolateral amygdaloid nucleus; BMA: Basomedial amygdaloid nucleus, anterior part; Au1: Primary auditory cortex.

The results revealed that, in the CSD group, brain regions exhibiting significant decreases in ALFF included S1BF, S1HL, S2, and CPU, while regions showing significant increases included the hippocampal CA2 region. In terms of ReHo, significant decreases were observed in GI, BMA, BLA, and CA1, while significant increases were found in S1BF, Au1, and CA2. Notably, both ALFF and ReHo values were significantly elevated in the CA2 region of the hippocampus (Figure 2). These findings suggest that the hippocampal CA2 region might play a critical role in the functional abnormalities induced by CSD, particularly in the context of spatial working memory.

### 3.3 Disrupted acetate metabolism in the hippocampus of CSD mice

Results of fMRI study have shown that CSD can lead to abnormal functionl activity in the hippocampus. The lack of sleep might reduce energy metabolism in this area, impairing its normal function, which in turn negatively impacts memory and learning abilities. To explore the mechanisms by which CSD affects spatial working memory from a metabolic perspective, we conducted a metabolic kinetics study.

**Figure 3:**
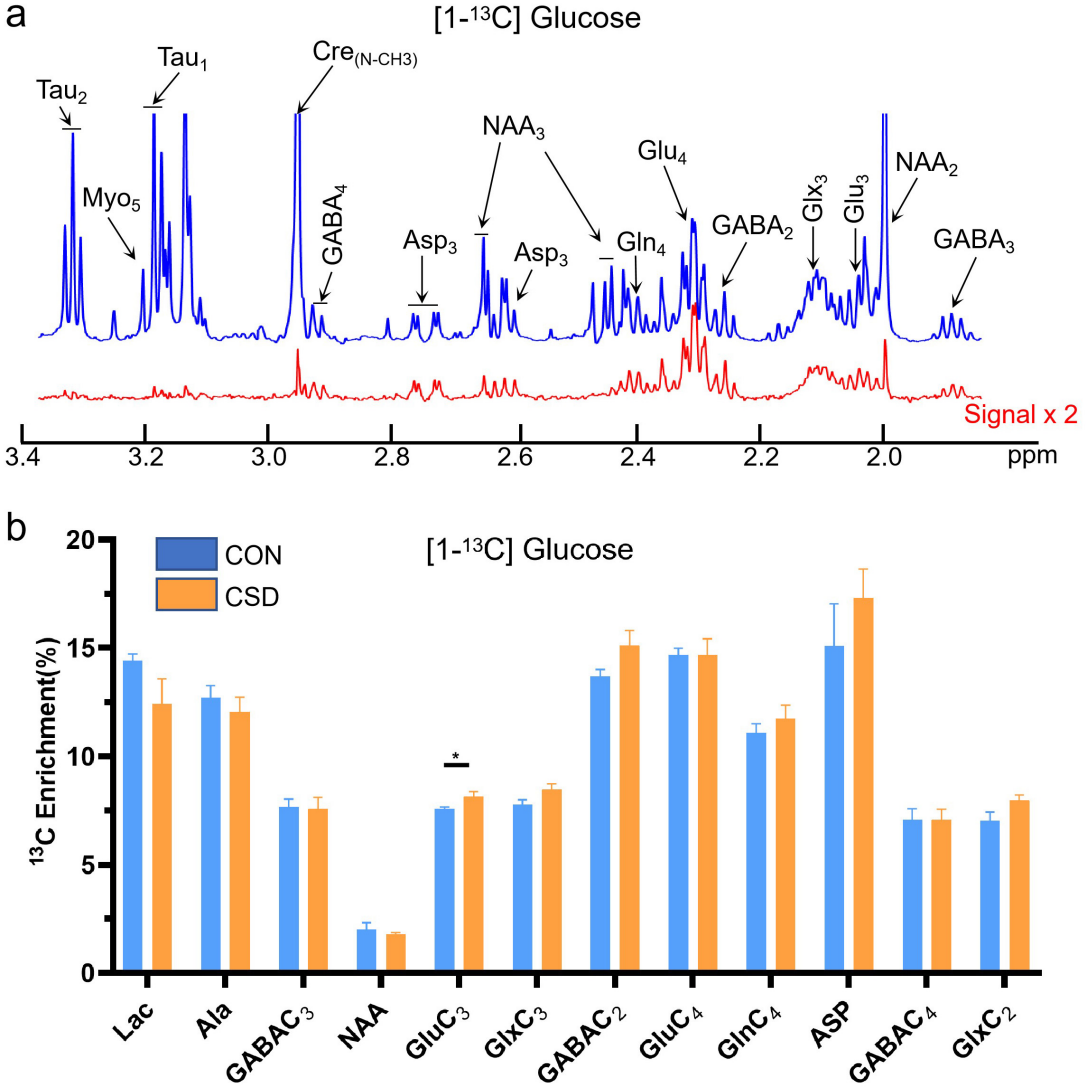
Glucose metabolism in the hippocampus of control and chronic sleep-deprived mice. **(a)** Representative NMR spectra of [1-13C] glucose. Blue line: NMR signals for all metabolites (12C + 13C), red line: Twice 13C-labeled metabolites. **(b)** Glucose metabolic differences in the hippocampus of mice. Lac: lactate; Ala: alanine; GABA: gamma-aminobutyric acid; NAA: N-acetylaspartate; Glu: glutamate; Glx: glutamine + glutamate; Gln: glutamine; Asp: aspartate; NMR: nuclear magnetic resonance; CON: Control group; CSD: Chronic sleep deprivation group. *p < 0.05, *n* = 7 mice per group.

**Figure 4:**
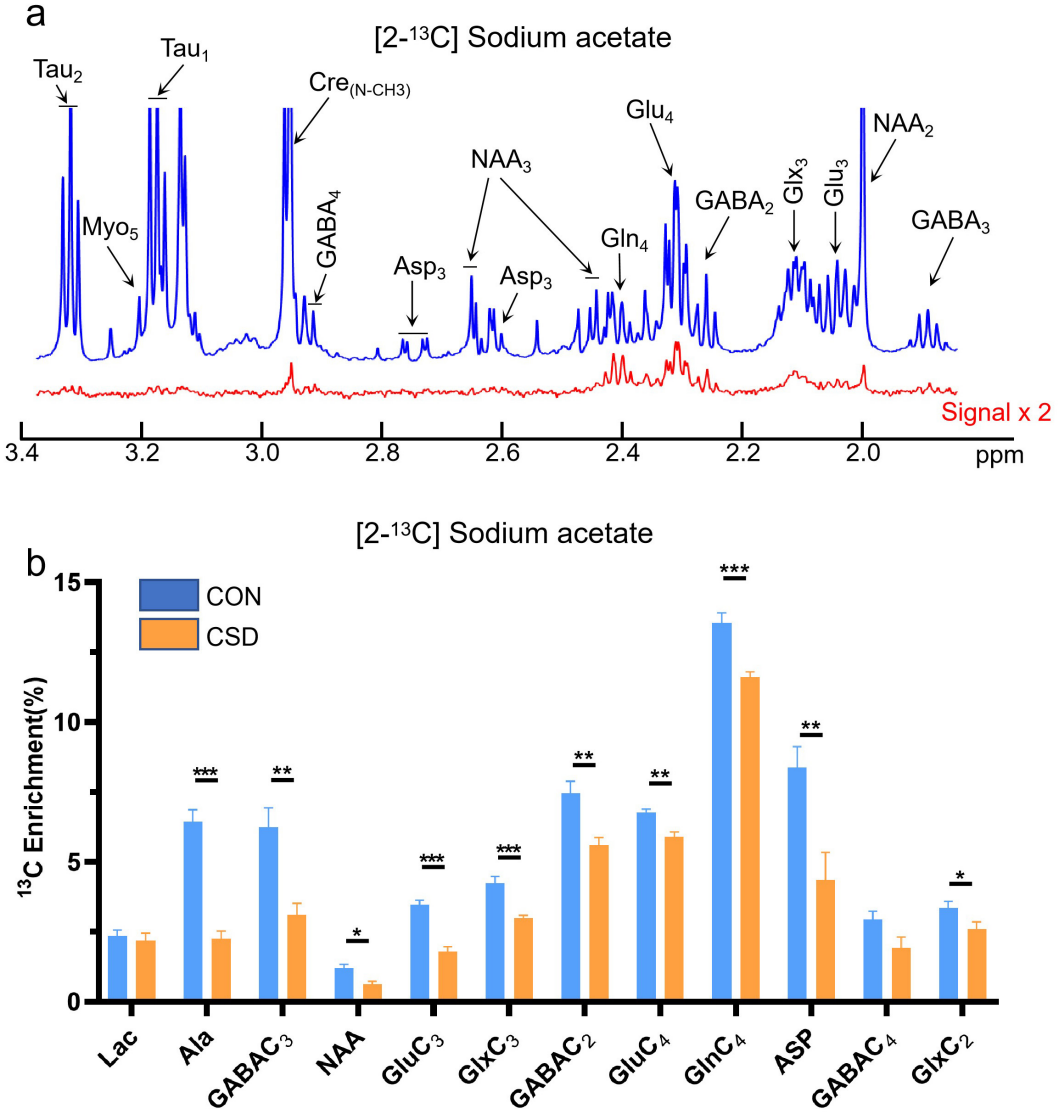
Sodium acetate metabolism in the hippocampus of control and chronic sleep-deprived mice. **(a)** Representative NMR spectra of [2-13C] Sodium acetate. Blue line: NMR signals for all metabolites (12C + 13C), red line: Twice 13C-labeled metabolites. **(b)** Sodium acetate metabolic differences in the hippocampus of mice. Lac: lactate; Ala: alanine; GABA: gamma-aminobutyric acid; NAA: N-acetylaspartate; Glu: glutamate; Glx: glutamine+glutamate; Gln: glutamine; Asp: aspartate; NMR: nuclear magnetic resonance; CON: Control group; CSD: Chronic sleep deprivation group. *p < 0.05, **p < 0.01, ***p < 0.001, *n* = 7 mice per group.

The metabolic changes of glucose in the hippocampus over a 30 min period were collected (Figure 3). Glucose serves as a direct energy source for neurons and can also be absorbed and utilized by glial cells (Magistretti and Allaman, 2015). Therefore, the observed glucose metabolism changes represent metabolic alterations in hippocampal neurons and astrocytes. Compared to the control group, only glutamate metabolism showed a significant increase [t (12) =2.29, *P* = 0.0357]. Other metabolites, however, did not show significant changes. When acetate is used as a nutrient, it is primarily absorbed and metabolized by astrocytes, serving as a widely accepted proxy for astrocytic metabolism *in vivo*. Our data reveal a decline in the levels of key astrocytic metabolites within the hippocampus, including lactate, glutamine, and aspartate. Notable decreases were observed in metabolites such as GABA3 [t (12) = 2.35, *P* = 0.031], NAA [t (12) = 2.85, *P* = 0.0109], Glu3 [t (12) = 5.04, *P* = 0.0001], Glx3 [t (12) = 4.75, *P* = 0.0002], Glu4 [t (12) = 3.85, *P* = 0.0013], Gln4 [t (12) = 4.32, *P* = 0.0005], and ASP3 [t (12) = 3.20, *P* = 0.0063] (Figure 4).

Glutamine, a primary metabolic product of astrocytes, plays a critical role in the glutamate-glutamine cycle, where it converts neuronal glutamate into glutamine, thereby maintaining metabolic balance in neurons. Under CSD, the metabolic activity of astrocytes is likely impaired, resulting in alterations to glutamine levels, which subsequently affects hippocampal metabolic function. While both neurons and astrocytes contribute to hippocampal metabolic regulation, the influence on astrocytic metabolism appears to be more pronounced in CSD models. Astrocytes are integral to the metabolic processes of various neurotransmitters, exerting significant effects on neuronal metabolic activities through pathways such as the glutamate-glutamine cycle. Consequently, astrocytes may play a more pivotal role in hippocampal metabolic regulation during CSD.

### 3.4 Fluorocitric acid disrupts astrocytic metabolism in CA2 region and impairs spatial memory

In the present study, we observed that CSD exerted a more profound effect on astrocytic metabolism in the hippocampus. Additionally, functional MRI co-marking of ALFF and ReHo revealed abnormal activity in the CA2 region of the hippocampus in CSD mice. Building on these findings, we aimed to investigate whether metabolic alterations in astrocytic within the CA2 region contribute to deficits in spatial memory.

Fluorocitric acid (Willoughby et al., 2003), a compound that inhibits mitochondrial oxidative phosphorylation in neurons, disrupts proton pump activity in the inner mitochondrial membrane by inhibiting citrate synthase in the tricarboxylic acid cycle, leading to a reduction in ATP production. Furthermore, fluorocitric acid impedes calcium ion channels in the mitochondrial membrane, resulting in dysregulated intracellular calcium concentrations, which directly impair astrocyte function. While fluorocitric acid is not readily absorbed by neurons, limiting its impact on neuronal activity, it interferes with the normal functioning of astrocytes, disrupting their regulatory roles in brain regions.

**Figure 5:**
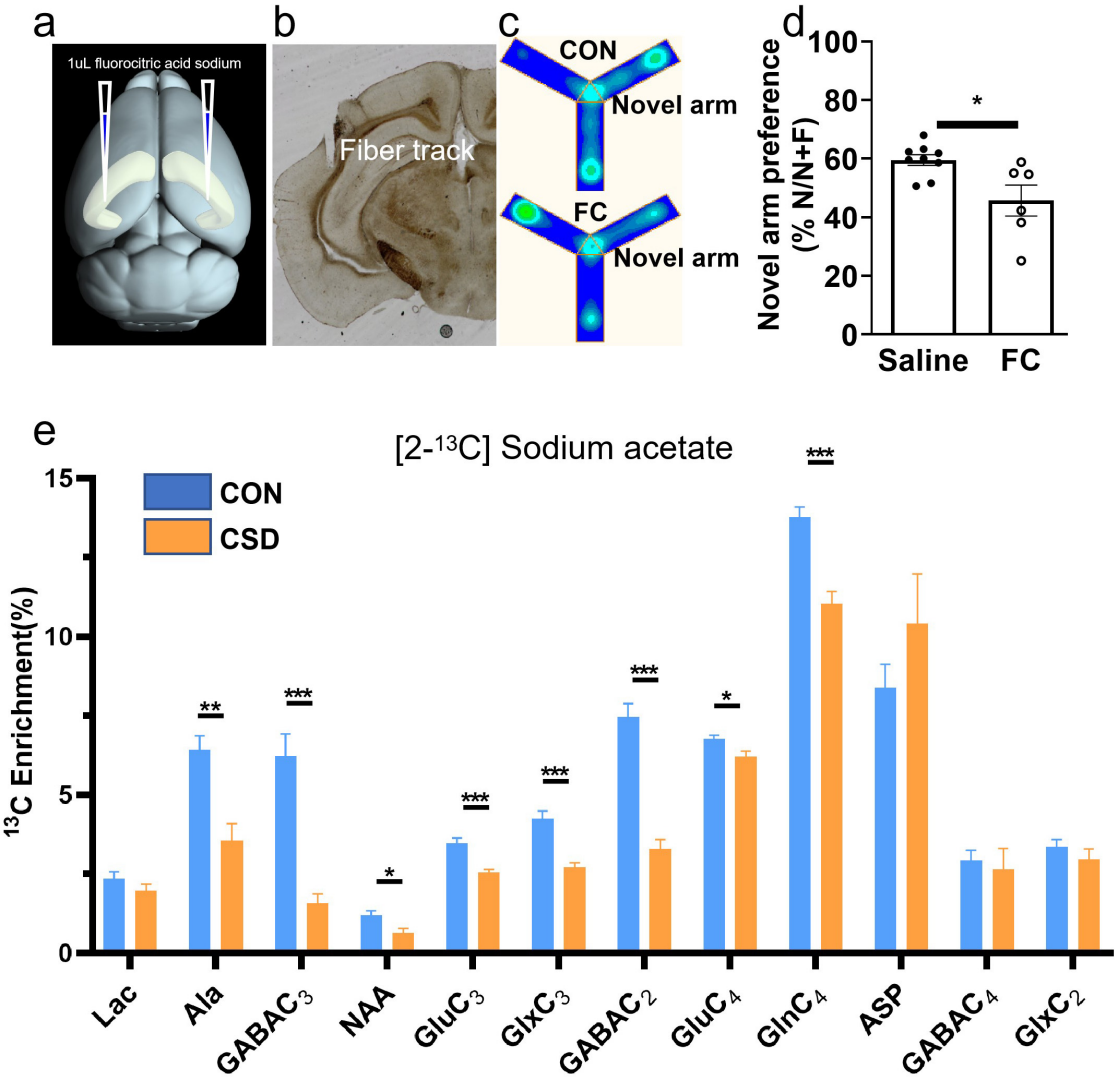
Cannula-delivered fluorocitric acid alters brain metabolism and behavior in mice. **(a)** Schematic of virus injection procedure. **(b)** Cannula trajectory within the CA2 region. **(c)** Heat map of Y-maze activity trajectories. **(d)** Y-maze performance in the control and cannula administration groups (CON group, *n* = 9 mice; FC group, *n* = 6 mice). **(e)** Fluorocitric acid injection into the CA2 region modulates metabolic dynamics of hippocampal astrocytes (n = 10 mice per group). Lac: lactate; Ala: alanine; GABA: gamma-aminobutyric acid; NAA: N-acetylaspartate; Glu: glutamate; Glx: glutamine+glutamate; Gln: glutamine; Asp: aspartate; FC: fluorocitric acid group; CON: Control group; CSD: Chronic sleep deprivation group. *p < 0.05, **p < 0.01, ***p < 0.001.

Following local injection of fluorocitric acid into the CA2 region via cannula (Figures 5a, b), we observed a significant reduction in hippocampal metabolic capacity, as evidenced by alterations in metabolites including Ala3 [t(18) = 3.30, *P* = 0.0026], GABA3 [t(18) = 4.59, *P* = 0.0001], NAA [t(18) = 2.80, *P* = 0.0093], Glu3 [t(18) = 3.80, *P* = 0.0008], Glx3 [t(18) = 4.10, *P* = 0.0003], GABA2 [t(18) = 4.59, *P* = 0.0001], Glu4 [t(18) = 2.28, *P* = 0.0351], and Gln4 [t(18) = 4.20, *P* = 0.0004] (Figure 5e). These results indicated that metabolic disruptions in astrocytes in the CA2 region propagate alterations across the hippocampus, underscoring the critical role of CA2 in maintaining metabolic stability within the hippocampus. The Y-maze task was conducted 30 mins following local injection into the CA2 brain region (Figure 5c). Mice injected with fluorocitric acid showed a significantly reduced ability to navigate the correct path compared to the saline-injected control group [t(13) = 2.65, *P* = 0.01] (Figure 5d), indicating a notable impairment in spatial memory. These findings suggest a critical relationship between astrocyte metabolism in the CA2 region and spatial memory function.

### 3.5 Inhibition of spontaneous calcium signaling in hippocampal CA2 astrocytes impairs spatial working memory in mice

We observed that metabolic dysfunction in hippocampal CA2 astrocytes induces spatial working memory impairments in mice. To further elucidate the underlying mechanisms, we employed viral tools to confirm these functional alterations and gain deeper insights into their cellular dynamics.

**Figure 6:**
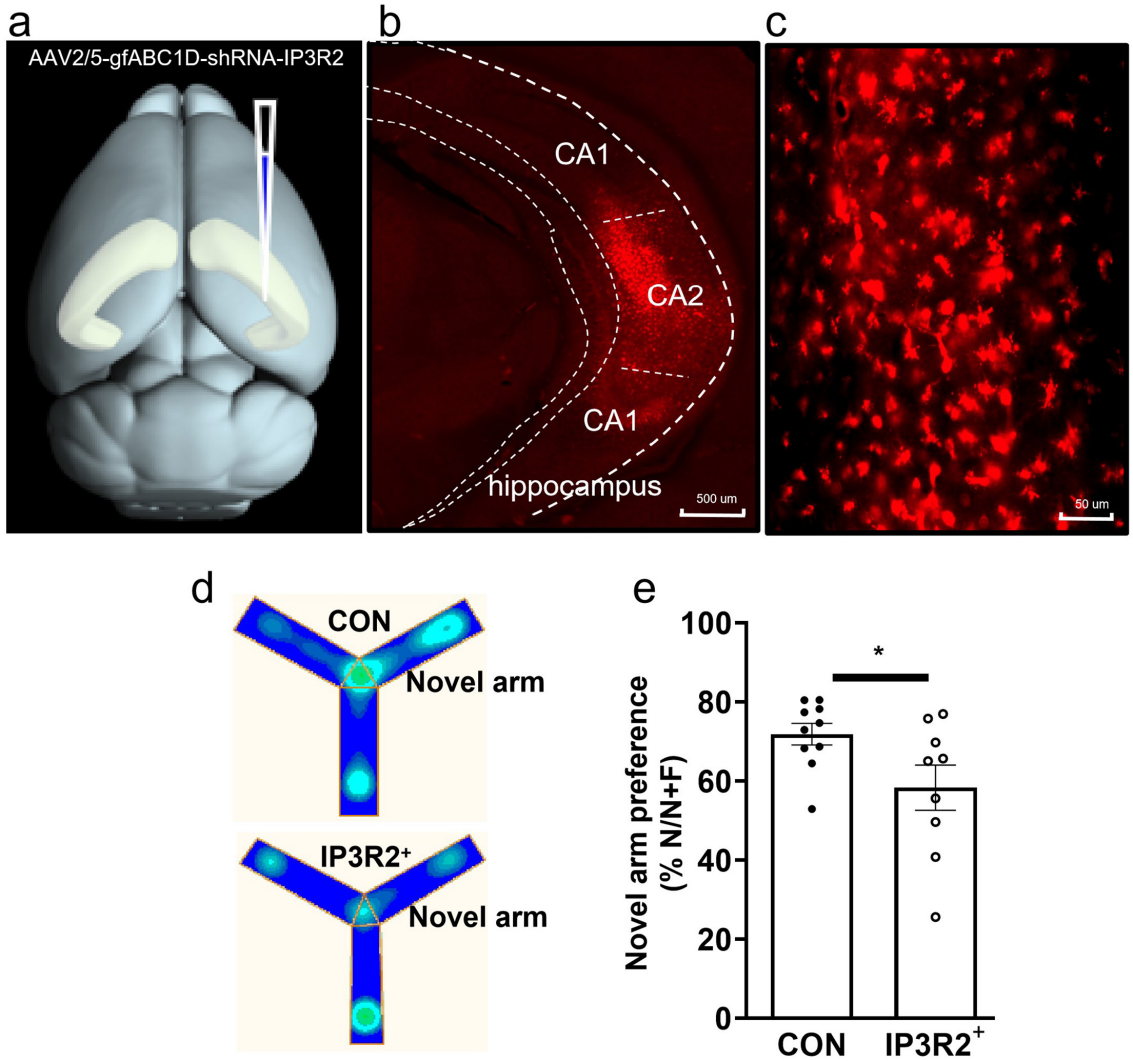
Inhibition of spontaneous calcium signals in the CA2 region impairs short-term spatial memory. **(a)** Diagram of virus injection procedure. **(b)** Viral expression within the hippocampus. **(c)** Viral expression in the CA2 region of the hippocampus. **(d)** Heatmap of Y-maze activity trajectories. **(e)** Y-maze performance in the control and viral groups. CON: Control group, *n* = 10 mice; IP3R2+: IP3R2+ group, *n* = 9 mice. ^*^p < 0.05.

Astrocytes play a critical role in regulating the extracellular environment surrounding neurons, including ion concentration and pH, to maintain neuronal homeostasis. Dysregulated astrocytic activity can destabilize this environment, thereby impairing the formation and expression of spatial memory. In terms of ion homeostasis, astrocytes maintain cellular ionic balance by modulating ion channels and transporters on the plasma membrane. Notably, calcium ions are involved in numerous cellular signaling and regulatory processes. By regulating the concentration of calcium ions both intracellularly and extracellularly, astrocytes influence cellular metabolism, signal transduction, and synaptic function. To investigate the role of astrocytic Ca^2+^ signaling in spatial working memory, we injected AAV2/5-gfABC1D-shRNA-IP3R2 into the hippocampal CA2 region of mice (Figures 6a–c). This approach used AAV2/5 as a vector to deliver the gfABC1D gene and shRNA targeting IP3R2 into astrocytes, thereby suppressing spontaneous calcium signaling in astrocytes via inhibition of IP3R2 expression. After 21 days infection, we conducted a Y-maze task, which revealed a significant reduction in the proportion of mice selecting the correct path following inhibition of astrocytic calcium signals [t(17) = 2.12, *P* = 0.04] (Figures 6d, e), indicating a marked decline in short-term memory. Previous studies have linked the hippocampal CA1 region to spatial perception and the CA3 region to long-term memory and spatial pattern recognition. The CA2 region, however, has been less well explored, despite its role as a relay station for memory transfer. Our findings suggest that spontaneous calcium activity in CA2 astrocytes is essential for spatial working memory.

### 3.6 Activation of hippocampal CA2 astrocytes restores spatial working memory in CSD mice

Our findings demonstrate that inhibiting astrocyte activity in the hippocampal CA2 region leads to significant impairments in spatial memory in mice. Building upon these results, we aimed to investigate whether activating astrocytes in the CA2 region could facilitate the recovery of spatial memory. To this end, we employed a chemogenetic approach to activate CA2 astrocytes and assess its effects on spatial memory performance.

**Figure 7:**
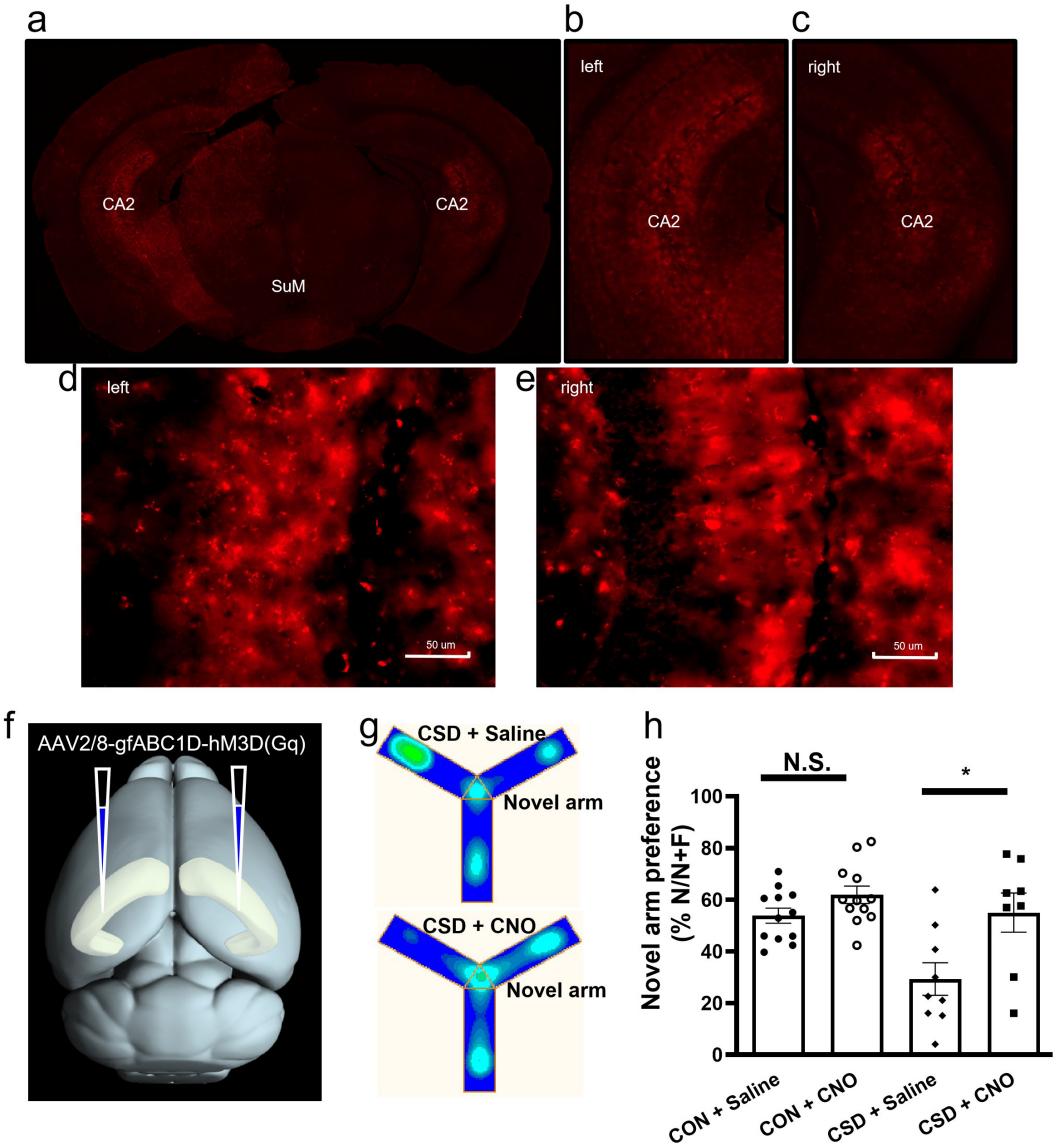
Activation of CA2 region astrocytes restores short-term spatial memory in sleep-deprived mice. **(a)** General viral expression. **(b)** Viral expression in the left CA2 region. **(c)** Viral expression in the right CA2 region. **(d)** Astrocytic infection in the left CA2 region. **(e)** Astrocytic infection in the right CA2 region. **(f)** Schematic of virus injection procedure. **(g)** Heatmap of Y-maze activity trajectories. **(h)** Y-maze performance before and after sleep deprivation in the control and CNO administration groups. CON: Control; CSD: Chronic sleep deprivation. CON+Saline: CON+Saline group, *n* = 12 mice; CON+CNO: CON+CNO group, *n* = 12 mice; CSD+Saline: CSD+Saline group, *n* = 9 mice; CSD+CNO: CSD+CNO group, *n* = 8 mice. ^*^p < 0.05.

We injected AAV2/8-gfABC1D-hM3Dq (Gq)-mCherry-ER2-WRPA-PE into the hippocampal CA2 region of mice (Figures 7a–f). This viral vector system allowed us to introduce the hM3Dq (Gq) receptor gene into astrocytes, with mCherry serving as a marker for successful gene expression. The hM3Dq (Gq) receptor is activated in the presence of clozapine-N-oxide (CNO), initiating the Gq signaling pathway, which regulates astrocyte activity. Twenty-one days post-injection, we conducted a Y-maze test to evaluate spatial memory performance (Figure 7g). In the initial phase of the experiment, we administered either CNO or saline to non-modeled (CSD) mice, followed by a Y-maze test 30 mins later. No significant differences in the proportion of mice correctly navigating the maze were observed between the CNO and saline-treated groups (Figure 7h), suggesting that activation of astrocytes in the absence of prior manipulation did not alter spatial memory performance. Subsequently, the mice underwent 14 days of CSD. As expected, spatial memory performance significantly declined in the sleep-deprived mice, with a marked reduction in the proportion of mice finding the correct path. Following this, we administered CNO intraperitoneally to activate astrocytes in the hippocampal CA2 region. Notably, astrocyte activation led to a significant improvement in spatial memory performance, with the proportion of mice correctly navigating the maze increasing [*F*_(3, 37)_ = 4.31, *P* = 0.01, df1 = 3, df2 = 37] (Figures 7g, h). This recovery in spatial memory performance was comparable to pre-sleep deprivation levels, indicating that astrocyte activation in the hippocampal CA2 region played a pivotal role in restoring spatial working memory. Taken together, these findings highlight the crucial role of hippocampal CA2 astrocytes in regulating spatial working memory, particularly under conditions of CSD, and suggest potential avenues for therapeutic intervention in memory dysfunction.

## 4 Discussion

Sleep deprivation has profound negative effects on spatial learning and cognition. The hippocampus, a key center for memory, plays an essential role in the formation and storage of spatial memories. While research on the mechanisms underlying chronic sleep deprivation-induced impairments in spatial learning and memory has largely focused on the dentate gyrus, CA1, and CA3 regions of the hippocampus (Wilson and McNaughton, 1994; Meier et al., 2020; Konakanchi et al., 2022), the CA2 region has received comparatively less attention. In this study, we employed functional magnetic resonance imaging (fMRI) to identify the brain regions implicated in the decline of spatial working memory following sleep disturbances at the whole-brain level. Resting-state fMRI analysis relies on two key parameters: the amplitude of low-frequency fluctuation (ALFF) and regional homogeneity (ReHo). ALFF is a measure of brain activity, with higher values indicating increased neural activity in a given region, while lower values reflect reduced neural activity (Zhang et al., 2021). ReHo, on the other hand, assesses local functional coherence, with areas exhibiting higher consistency being considered more stable and coordinated, and those with lower consistency suggesting neural instability or heterogeneity. The combined analysis of ALFF and ReHo offers a direct assessment of functional changes in target brain regions. It is worth noting that regarding the observed hemispheric asymmetry, such subtle differences in fMRI signals may arise from individual variability in postural preferences or localized physiological fluctuations during the sleep deprivation protocol. By employing bilateral ROI analysis, we aimed to capture the overall functional state of the CA2 region, ensuring that our conclusions reflect the general impact of chronic sleep deprivation on hippocampal circuitry. Using this approach, we identified the CA2 region of the hippocampus as a critical brain area for spatial memory.

The CA2 region, located centrally within the hippocampus, has a less defined role in spatial memory compared to the surrounding CA1 and CA3 subregions. However, growing evidence underscores its importance in spatial learning and memory formation (Cui et al., 2013; Chang et al., 2023; Fredes et al., 2021). For instance, a study led by Massadeh et al. identified that abnormalities in spatial memory within a sleep deprivation model correlate with altered metabolic activity in the hippocampus (Massadeh et al., 2022). The observed increase in ALFF and ReHo values in the CA2 region, occurring alongside decreased astrocytic metabolism, highlights a state of neuro-metabolic uncoupling induced by CSD. We propose that the elevated fMRI signals represent a compensatory hyperactivation, where the CA2 circuitry exhibits pathologically increased spontaneous activity and regional synchronization in an inefficient attempt to maintain functional output despite metabolic stress (Peng et al., 2024). This “high activity-low metabolism” paradox suggests a failure in neurovascular coupling: while the BOLD-fMRI signal (reflecting local blood flow and oxygenation) increases due to aberrant neural firing, the underlying astrocytic metabolic support—specifically the supply of energy substrates like lactate—is significantly compromised (Iadecola, 2017). Such uncoupling indicates that the CA2 region is operating in a metabolically unsustainable state, which ultimately leads to the failure of spatial working memory consolidation.

Similarly, we conducted metabolic kinetic studies using 13C-labeled glucose and sodium acetate to assess neuronal and astrocytic function. Our findings indicate that astrocytic metabolism in the hippocampus is disrupted in sleep-disordered mice. The metabolic changes in the hippocampus induced by sleep deprivation involve complex interactions between neurons and astrocytes. Neurons, particularly through glutamate, an excitatory neurotransmitter, play a central role in cognitive functions, including learning and memory (Meldrum, 2000). In the context of sleep deprivation, increased neuronal activity likely elevates glutamate release, contributing to higher glutamate levels within the hippocampus. However, it is important to recognize the role of astrocytes in glutamate metabolism (Dienel, 2019). Astrocytes participate in the glutamate-glutamine cycle, converting glutamate into glutamine, which may have a more significant impact on metabolic regulation in the hippocampus. Moreover, glucose metabolism encompasses both neuronal and glial processes, which could counterbalance one another. This may explain why metabolic products of glucose, such as GABA and N-acetylaspartate (NAA), showed no significant changes in our study.Furthermore, our findings reveal a critical divergence between neuronal and astrocytic metabolic pools. In the glucose tracer study, metabolites such as GABA and NAA showed no significant change, suggesting that the primary neuronal metabolic reservoirs remain relatively stable under CSD. In stark contrast, the acetate tracer study revealed a significant reduction in these same metabolites (GABA, *P* = 0.031, df = 12; NAA, *P* = 0.0409, df = 12). This selective impairment in acetate-derived metabolites provides direct evidence of specific astrocytic dysfunction and a failure in the metabolic support system that astrocytes provide to neurons.

Our finding of decreased acetate metabolism in the CA2 region following CSD provides a complementary perspective to previous reports of increased brain lactate levels during sleep deprivation (Naylor et al., 2012). While elevated lactate is often interpreted as a compensatory response to meet the increased energy demands of the waking brain, our data using ^13^C-labeled acetate reveals a specific impairment in the astrocytic metabolic machinery itself. The reduction in acetate-derived metabolites suggests that despite a potential abundance of fuel (such as lactate), the ability of CA2 astrocytes to process these substrates through the TCA cycle and the glutamate-glutamine cycle is compromised. This distinction highlights that CSD-induced cognitive deficits may stem not just from energy depletion, but from a fundamental failure in astrocytic metabolic support and recycling capacity.

In this study, we demonstrate that inhibiting astrocytic metabolism in the CA2 region disrupts spatial working memory in mice. We propose several potential mechanisms underlying this effect. First, astrocytes are crucial for maintaining neuronal function and metabolic homeostasis (Gibbs, 2016). Inhibition of astrocyte metabolism in CA2 may alter the hippocampal microenvironment, reduce neuronal activity, and impair spatial memory. Second, astrocytes regulate energy metabolism by modulating glucose and lactate metabolism, supplying essential energy to neurons (Herrera Moro Chao et al., 2022). Consequently, inhibiting astrocyte metabolism in CA2 could disrupt the delivery of glucose and lactate, compromising neuronal energy metabolism, diminishing energy supply, and impairing neuronal function, thus affecting spatial memory formation and storage. Third, astrocytes contribute to synaptic signaling by regulating neurotransmitter synthesis and clearance (Kofuji and Araque, 2021). Inhibition of astrocyte metabolism may interfere with neurotransmitter dynamics, leading to aberrant signal transmission between neurons, which disrupts neuronal network activity and synaptic function, ultimately impairing spatial working memory. Fourth, astrocytes maintain acid-base balance and water homeostasis, which are vital for normal neuronal function (Mishra, 2017). Inhibiting astrocytic metabolism may destabilize the extracellular environment by disturbing acid-base balance and water content, thereby affecting neuronal function and synaptic activity, with subsequent impacts on spatial memory.

Recent research suggests that spontaneous calcium activity in hippocampal CA2 astrocytes is a critical feature for short-term memory processes. This spontaneous calcium signaling in CA2 astrocytes contributes to the transmission and processing of information by modulating the strength of synaptic connections between neurons, ultimately influencing the formation and storage of short-term spatial memory. Inositol trisphosphate (IP3), a second messenger molecule, regulates intracellular calcium ion concentrations, affecting neuronal excitability and synaptic transmission efficiency (Sherwood et al., 2021), thus playing a direct role in memory formation and storage. Following inhibition of IP3-mediated calcium signaling in CA2 astrocytes, mice exhibited a decline in spatial memory performance, highlighting the importance of IP3 released from hippocampal CA2 astrocytes in spatial memory. This effect may stem from the involvement of the IP3 signaling pathway in regulating synaptic plasticity (Choi et al., 2018), thereby influencing synaptic connections and transmission between CA2 neurons, which in turn affects spatial memory. Furthermore, IP3 is implicated in astrocytes' regulation of the neuronal microenvironment. Dysregulation of this environment can disrupt neuronal activity. Therefore, IP3 abnormalities may also indirectly affect hippocampal CA2 neuronal activity patterns and network oscillations, further compromising spatial memory formation and retrieval (Semyanov, 2019).

A key question arises regarding the internal link between the observed metabolic disruption and calcium signaling abnormalities in CA2 astrocytes. We propose that these two phenomena are mechanistically coupled through the regulation of cellular energy status and endoplasmic reticulum (ER) calcium homeostasis. Astrocytic metabolism, particularly the acetate-fueled TCA cycle, is essential for maintaining local ATP levels. Since the Sarco/Endoplasmic Reticulum Ca2+-ATPase (SERCA) pump—responsible for refilling ER calcium stores—is highly energy-dependent, a deficit in astrocytic ATP production following CSD may impair ER calcium sequestration (Horvat et al., 2021). Consequently, the depletion of ER calcium stores would suppress IP3R2-mediated spontaneous calcium oscillations, which are the primary source of the calcium signaling deficits observed in our study (Vardjan et al., 2017; Bonvento and Bolaños, 2021). This integrated model suggests that metabolic failure acts as an upstream driver that compromises astrocytic calcium signaling, ultimately leading to the impairment of spatial working memory.

In this study, we found that astrocytes in the hippocampal CA2 region are closely linked to spatial working memory in mice. By activating calcium signaling in CA2 astrocytes, we were able to mitigate the effects of sleep deprivation on spatial working memory. Synaptic plasticity, which involves the modulation of synaptic strength between neurons, is a key mechanism in memory formation and storage (Lee et al., 2023). Therefore, activating CA2 astrocytes likely enhances synaptic plasticity, making the neural circuits involved in spatial memory more susceptible to long-term changes following learning, thereby improving spatial working memory storage. Additionally, activation of the CA2 calcium pathway facilitates information processing and integration. CA2 astrocytes may promote memory storage by enhancing these processes (Araque, 2008). This activation can lead to increased neuronal activity, thereby boosting the transmission and processing of information within neural circuits (Perea and Araque, 2006). As a result, spatial memory-related information is more effectively processed and integrated into the brain's memory network, strengthening the stability and reliability of short-term memory storage.

This study also have limitation. We acknowledge the critical importance of cell-type specificity in viral manipulations. While our histological evidence (Figure 6b) confirms that the gfABC1D promoter provides high selectivity for CA2 astrocytes, we recognize the inherent limitations of promoter-only systems, which can exhibit minor neuronal leakage. As highlighted by the recent work of Gleichman et al. (2023), the integration of microRNA-targeting cassettes (such as miR-124 sequences) into AAV vectors represents a significant advancement in achieving absolute cell-type specificity (Gleichman et al., 2023). By leveraging the endogenous microRNA environment of neurons to degrade off-target transcripts, this approach could further refine the precision of astrocytic modulation in future studies.

In summary, we employed functional magnetic resonance imaging (fMRI) coupled with a metabolic dynamics model to investigate the effects of sleep deprivation on spatial working memory, identifying the hippocampal CA2 region as a pivotal neural hub. Further analysis revealed that astrocytes within the CA2 region play a critical role in modulating the impact of sleep deprivation on spatial memory. Specifically, inhibition of calcium signaling in CA2 astrocytes resulted in a marked impairment of short-term spatial memory, whereas activation of these astrocytes mitigated the cognitive deficits induced by sleep deprivation. These findings offer essential mechanistic insights into the regulatory function of the hippocampal CA2 in sleep-related memory disturbances and present a novel framework for future investigations into the pathways through which sleep deprivation disrupts spatial working memory.

## Data Availability

The data supporting the findings of this study are included in the article. Any additional data or information required for reanalysis are available from the lead contact upon request (jie.wang@shsmu.edu.cn, zhy12015@rjh.com).
